# Current knowledge on epidemiology and evolution of novel porcine circovirus 4

**DOI:** 10.1186/s13567-022-01053-w

**Published:** 2022-05-31

**Authors:** Dongliang Wang, Jinhui Mai, Yi Yang, Chao-Ting Xiao, Naidong Wang

**Affiliations:** 1 Hunan Provincial Key Laboratory of Protein Engineering in Animal Vaccines, Laboratory of Functional Proteomics (LFP), Research Center of Reverse Vaccinology (RCRV), College of Veterinary Medicine, Hunan Agricultural University, Changsha, 410128 China; 2 Institute of Pathogen Biology and Immunology, College of Biology, Hunan University, Changsha, China

**Keywords:** PCV4, Cap, epidemiology, genetic diversity

## Abstract

Porcine circovirus type 4 (PCV4) is a newly emerging virus, with both PCV4 genomic DNA and specific antibodies detected in swine herds in several provinces in China and South Korea. Although the virus was first identified in 2019 in Hunan, China, retrospective research suggests that serum samples collected as early as 2008 were positive for PCV4 antibody. Infections with only PCV4 or co-infections with other pathogens have been associated with several clinical manifestations, but its pathogenesis remains to be determined. The purpose of this review was the following: (1) to characterize PCV4 epidemiology by assessing evolutionary dynamics and genetic diversity of PCV4 strains circulating in swine herds; (2) to reconstruct a computerized 3D model to analyze PCV4 Cap properties; (3) and to summarize the current evidence of PCV4-associated clinical-pathological manifestations. The origin of PCV4 is apparently distinct from other PCV, based on analysis of phylogenetic trees. Of note, PCV4 shares an ancient common ancestor with mink circoviruses. Furthermore, the amino acid residue at position 27 of the PCV4 Cap is a key benchmark to distinguish PCV4a (^27^S) from PCV4b (^27^ N), based on PCV4 strains currently available, and variation of this residue may alter Cap antigenicity. In addition, the capsid surface of PCV4 has characteristics of increased polar residues, compared to PCV2, which raises the possibility that PCV4 may target negatively charged host receptors to promote virus infection. Further studies are required, including virus isolation and culture, and more detailed characterization of molecular epidemiology and genetic diversity of PCV4 in swine herds.

## Introduction

Porcine circoviruses (PCV) are members of the *Circovirus* genus in the *Circoviridae* family, characterized as non-enveloped viruses composed of a circular, single-stranded genomic DNA within an icosahedral capsid, ~17 nm in diameter [1–3]. To date, there are four identified types: PCV1, PCV2, PCV3, and a novel PCV4.

PCV4, a newly emerging circovirus, was first identified in 2019 in Hunan, China, in pigs with several clinical disease syndromes, including respiratory and enteric signs as well as porcine dermatitis nephropathy syndrome (PDNS) [3]. However, in retrospective studies, PCV4 genomic DNA was detected in swine tissue samples collected in 2012 from Henan, China [4], with some serum samples collected as early as 2008 from Chinese swine positive for PCV4 antibody [5]. Therefore, there is evidence that PCV4 has been present and circulating in swine herds for more than a decade.

**Figure 1:**
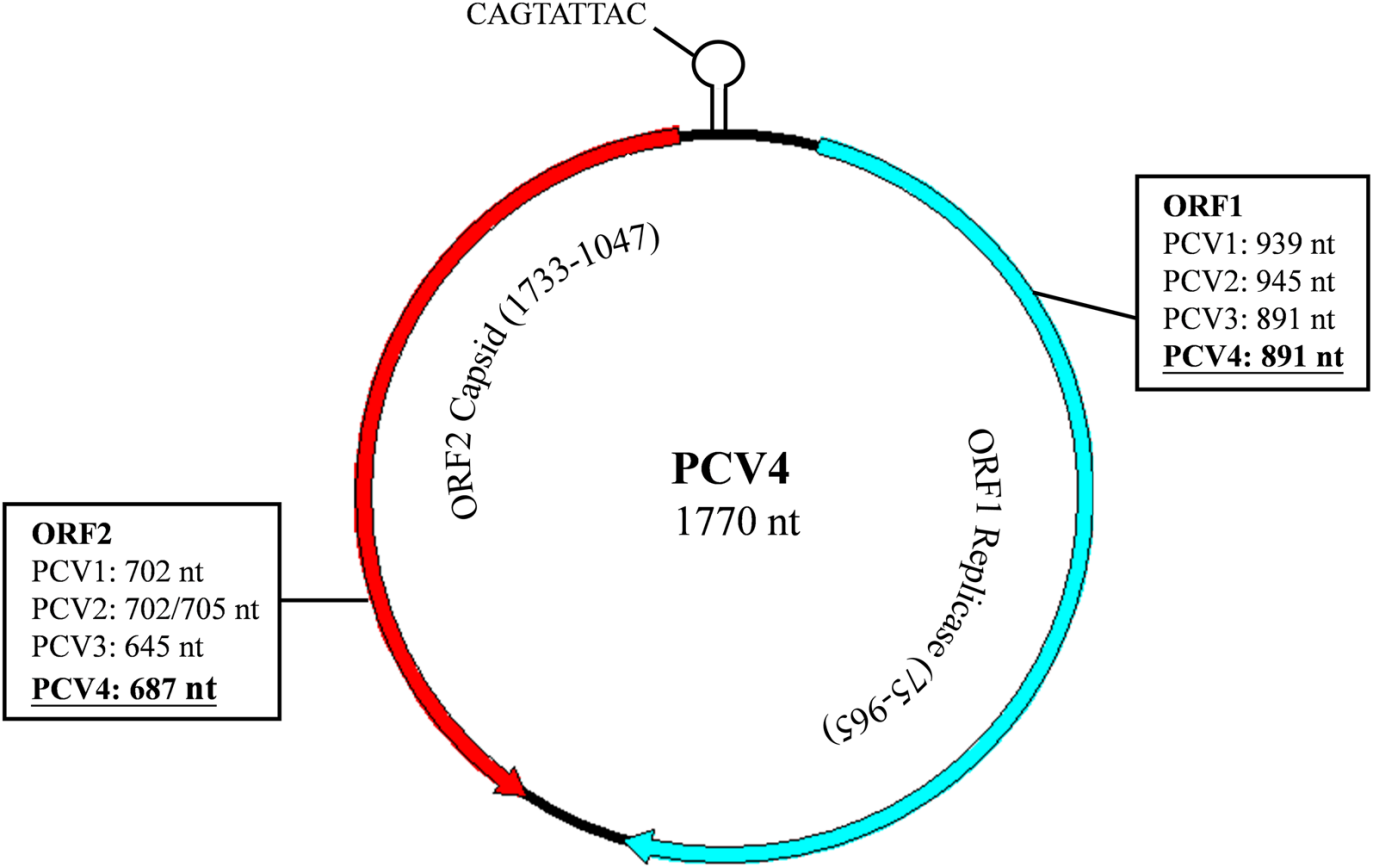
**Genomic characterization of PCV4.** The PCV4 genome, a single-stranded circular DNA genome with 1770 nt, contains two major ORF that differ from those in PCV1, PCV2 and PCV3. However, the stem-loop of PCV4 has a conserved 9-nt nonanucleotide sequence (CAGTATTAC) located within the intergenic region between ORF1 and ORF2.

The genome of PCV4 contains 1770 bases and a palindrome stem-loop structure, with the conserved nonanucleotide (CAGTATTAC) located within the intergenic region between two major open reading frames (ORF) (Figure 1). PCV4 has high nucleotide identity (66.9%) to mink circovirus, but low identities (43.2–51.5%) to other PCV [3]. ORF1 encodes the replicase protein (Rep) and the length of PCV4 ORF1 sequences differs from PCV1 and PCV2. In that regard, whereas ORF2 encodes the capsid protein (Cap), the length of PCV4 ORF2 sequences differs among PCV1, PCV2 and PCV3. Alignment of Cap sequences revealed that PCV4 has low identities with PCV1, PCV2 and PCV3 (~43.1, 45 and 24.5%, respectively) [3]. Cap is the sole structural protein of PCV, with a vital role in clathrin-mediated endocytosis, and actin- and small GTPase-dependent pathways for virus cell entry into host cells, as determined in a study with PCV2 [6, 7]. Additionally, it is noteworthy that Cap mutations cause antigenic drifts and potentially enable PCV2 and PCV3 to evade immunity [8, 9]. Evolutionary pressures driving mutations may enable the virus to generate resistance to antiviral treatment, evade host immune responses, and facilitate its adaptation to the environment and hosts. Thus, elucidating the evolutionary dynamics of Cap is key to understanding this emerging PCV4.

The purpose of this review was the following: (1) to characterize PCV4 epidemiology by assessing evolutionary dynamics and genetic diversity of PCV4 strains circulating in swine herds; (2) to reconstruct a computerized 3D model to analyze PCV4 Cap properties; and (3) to summarize clinical diseases associated with PCV4 infection.

## Evolution and genetic diversity of PCV4

### Evolutionary changes

Although PCV4 was discovered in 2019 in the Hunan province of China, retrospective studies demonstrated that PCV4 DNA was present in swine samples collected in 2012 [4], implying PCV4 has been circulating in pigs for at least a decade. Since 2019, PCV4 has been reported in several provinces of China, including Hunan, Henan, Jiangsu, Anhui, Shanxi, Guangxi, and Inner Mongolia [3, 4, 10–14]. Furthermore, PCV4 was also detected in domestic swine in Korea [15, 16]. Notwithstanding, based on failure to detect PCV4 DNA in swine samples (sera and tissues) from Europe (Italy and Spain) [17], it seems PCV4 appears to have a limited geographic distribution. Thus, distribution of PCV4 in other geographic regions requires further study.

**Figure 2:**
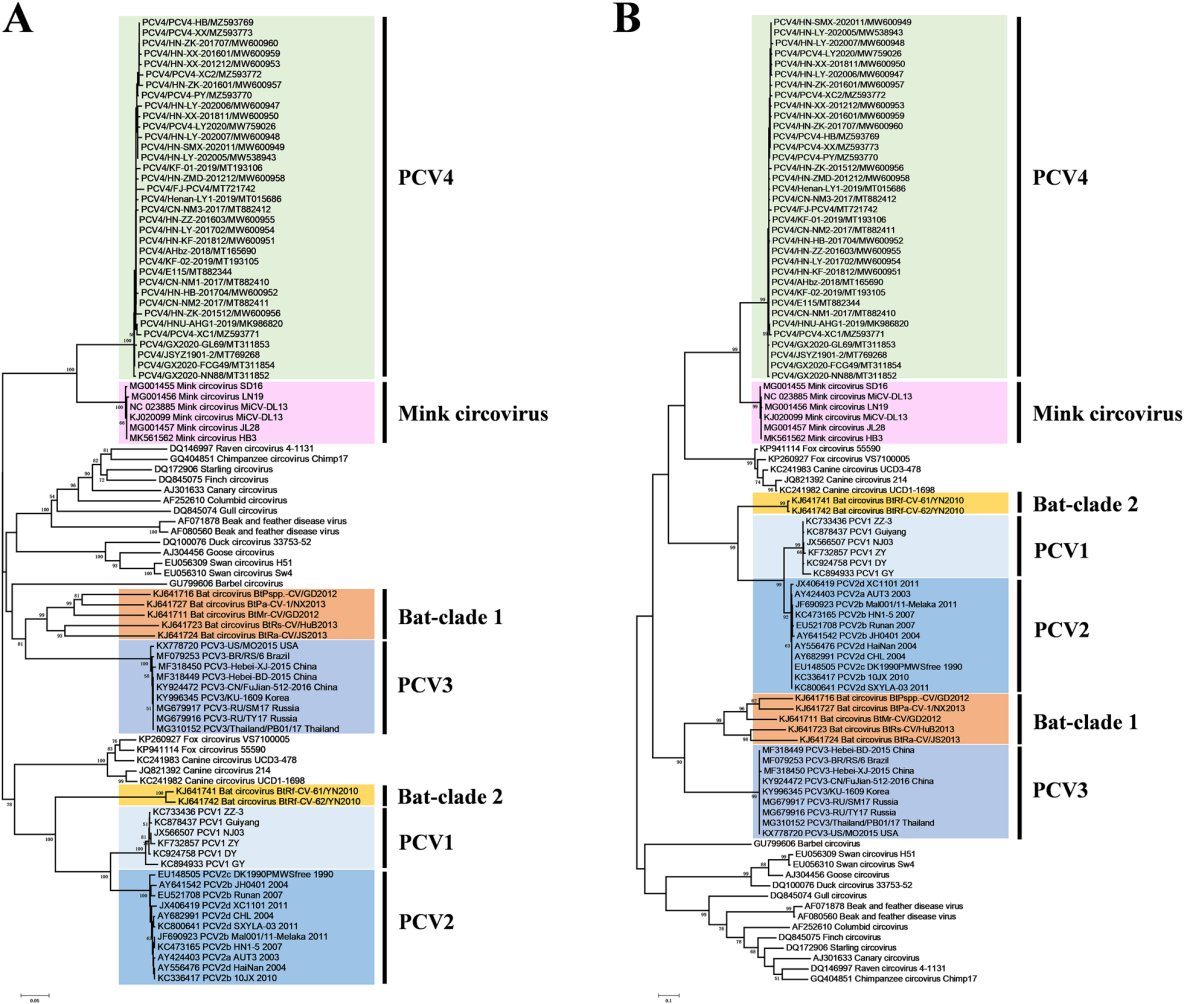
**Phylogenetic trees of circoviruses, using conserved Rep amino acid sequences.** The analysis contained 93 amino acid sequences. Neighbor joining (**A**) and maximum likelihood (**B**) trees were reconstructed using the p-distance and Jones-Taylor-Thornton (JTT) model, respectively, with 1000 bootstrap replicates and bootstrap >50%.

For a better understanding of the evolutionary origin of PCV4, we analyzed a dataset that included sequences from 35 PCV4 and other circoviruses from a variety of host species. RDP4 recombination analysis software was used to detect gene recombination, but there was no evidence of recombination. Sequences were aligned with the Clustal W method conducted in MEGA 7. To trace the origin of PCV, a phylogenetic tree was reconstructed, based on Rep amino acid sequences, using the neighbor joining (NJ) and maximum likelihood (ML) methods. Based on phylogenetic trees, we inferred that PCV1 and PCV2 are closely related to bat-clade 2 circoviruses, whereas PCV3 is closely related to bat-clade 1 circoviruses, indicating a potential bat circovirus origin (Figure 2). However, PCV4 has a distinct origin from other PCV (Figure 2). All PCV4 strains were closely related to mink circoviruses (NJ tree bootstrap = 1.0, ML tree bootstrap = 0.99). The NJ and ML trees had similar topology, emphasizing that PCV4 shares a common ancient ancestor with mink circoviruses, providing evidence that it is reasonable to trace the origin of PCV based on the conserved Rep protein.

### Genetic analysis

**Figure 3:**
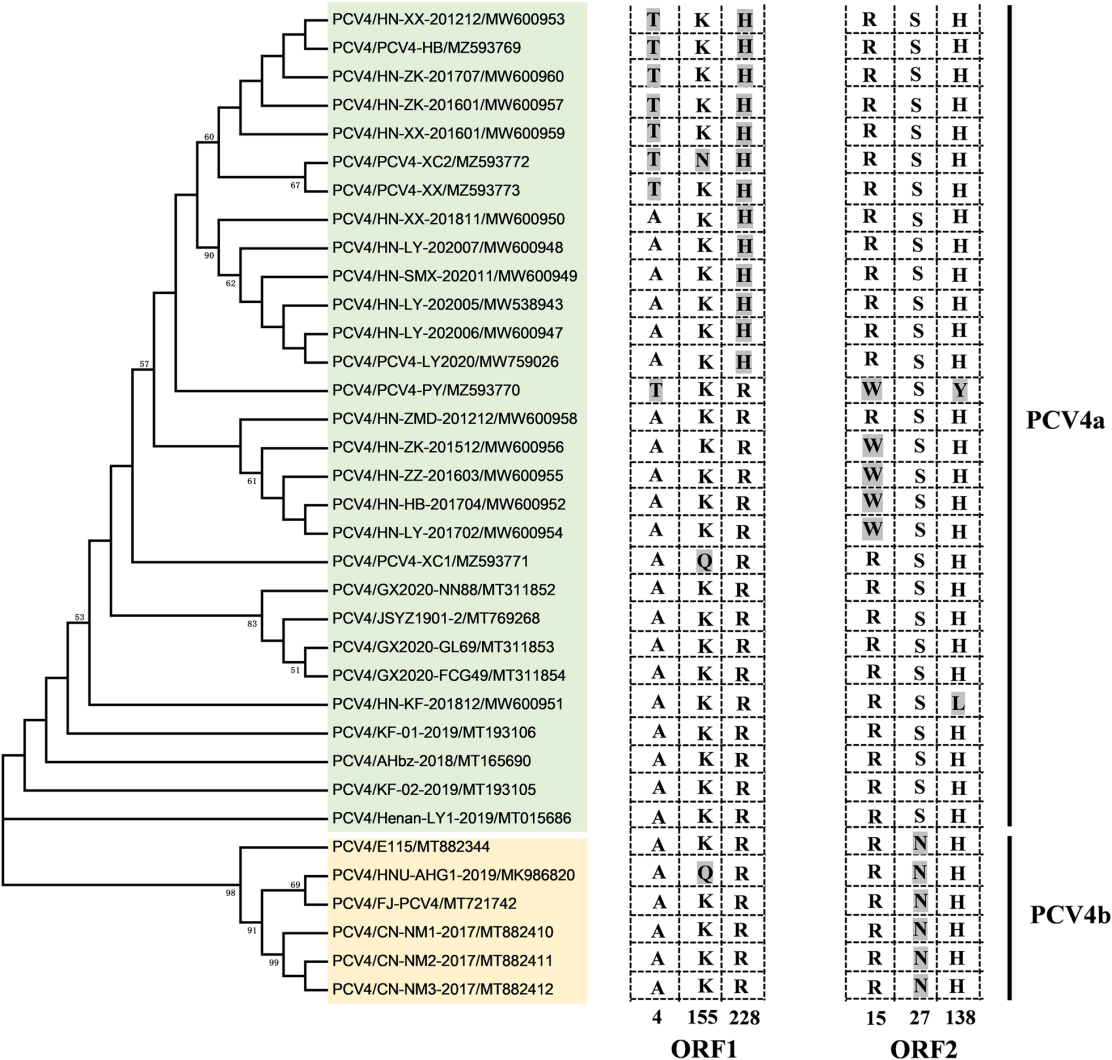
**Phylogenetic trees of PCV4, based on the complete genome.** A neighbor joining tree was reconstructed using p-distance model with 1000 bootstrap replicates and bootstrap >50%. Amino acid sequences of putative ORF1 and ORF2 were aligned with the ESPript 3.0 online tool, and the hot-spots of mutations are represented by the columns with their corresponding positions.

Based on complete genomes, *rep* or *cap* genes of all the 35 PCV4 sequences deposited in GenBank, phylogenetic trees were constructed, and PCV4 was divided into 2 temporary genotypes: PCV4a and PCV4b [18]. PCV4a contained 29 sequences collected from 2012 to 2021, whereas PCV4b only included 6 PCV4 sequences from 2017 to 2020. To better understand differences between the two genotypes, sequence alignments revealed that residues at positions 4, 155 and 228 in ORF1 and residues at positions 15, 27 and 138 in ORF2 were mutation hot-spots (Figure 3).

It is noteworthy that two-point mutations (R15W and S27N) occur in the putative nuclear localization signal (NLS) region of the PCV4 Cap, an arginine-rich region within the *circovirus* genus, which may be implicated in the package of the viral genome [15, 19]. Recently, it was reported that residue 1–20 of NLS is responsible for nucleolar localization of PCV4 Cap. Furthermore, the Cap is capable of directly interacting with nucleolar phosphoprotein nucleophosmin-1 (NPM1) [20]. The positively charged NLS remains buried in the internal surface of the PCV2 capsid, which may externalize in the metastable capsid during viral “breathing” [19]. Nevertheless, our previous study revealed that the peptide containing residues 1–17 of PCV2 NLS (defined NLS-A) was rapidly internalized via direct translocation by increased membrane permeability during cellular uptake [21]. Additionally, the positively charged residue (R) of PCV2 NLS-A changed to an uncharged residue (A), which significantly decreased membrane permeability and inhibited viral entry into cells (unpublished data). Thus, it was predicted that the mutation of R15W may affect PCV4 NLS functionality [18], although effects on membrane permeability remain to be determined. At position 27, a polar residue (^27^S) was changed to a similar polar one (N). Of note, the residue at position 27 was a critical site to distinguish PCV4a (^27^S) from PCV4b (^27^ N), based on all PCV4 sequences deposited in GenBank (Figure 3). Furthermore, epitope prediction suggests that the NLS contained a potential linear B cell epitope (5–35 aa). Furthermore, a previous study revealed that an epitope (26–36 aa) within the NLS of the PCV2 Cap, is a critical B cell epitope capable of eliciting neutralization antibody against PCV2 infection [22]. Therefore, mutations occurring at the two sites (15 and 27) of the NLS may alter antigenicity of the PCV4 Cap. However, potential immunogenic changes due to variations of the Cap NLS between PCV4a and PCV4b remain to be determined.

## Analysis of PCV4 Cap properties

**Figure 4:**
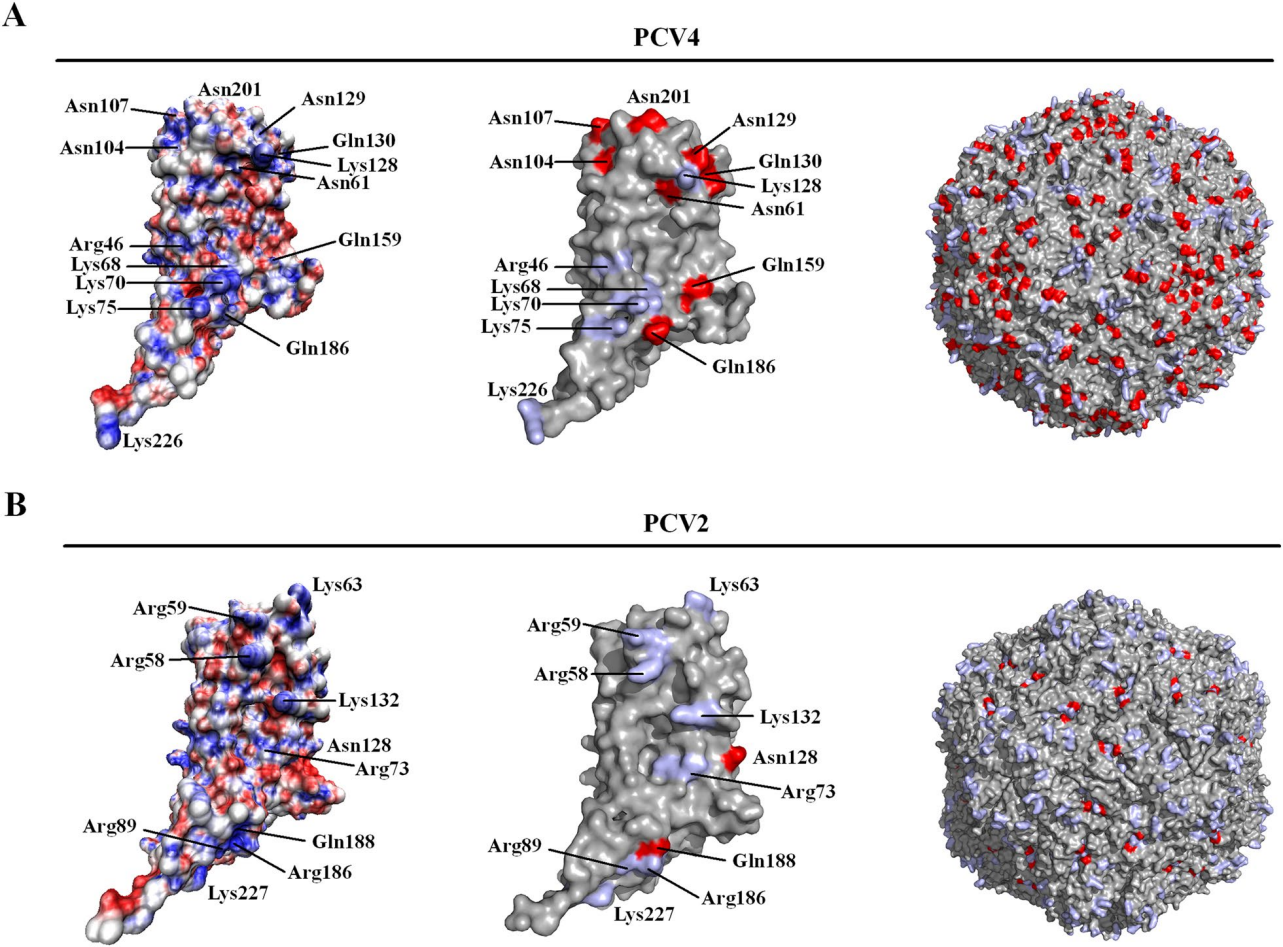
**3D structures of PCV2 and PCV4 Cap and capsid.** The 3D structure of PCV4 Cap was generated via homology modeling, using the PCV2 Cap as a template (PDB ID: 3R0R). 3D structures of Cap and capsid were displayed with Pymol version 1.8.0.3. Electrostatic surface analysis of Cap monomer (far left) was computed in Chimera. The positively charged and polar amino acid residues of the Cap monomer (center) and capsid (far right) were indicated by light blue and red, respectively.

The Cap is the sole structural protein, capable of eliciting robust immune responses and regarded as the major target antigen for PCV4 serological diagnosis and vaccine design [23]. Since the three-dimensional (3D) structure of PCV4 Cap has not been resolved, we modeled the 3D structure of the PCV4 Cap via homology modeling based on PCV2 Cap structure (PDB ID: 3R0R) in the SWISS-MODEL, as described [23]. There was a typical jelly-roll structure in the PCV4 Cap, although surface-exposed loops were distinctly different between PCV2 and PCV4, similar to the PCV2 Cap [23]. As a non-enveloped virus, the Cap has a vital role in entry of the virus into cells. PCV2 enters host cells via attachment of the PCV2 icosahedral capsid to the moiety of heparan sulfate (HS) or chondroitin sulfate B (CSB) on the cell surface [24]. Residues responsible for binding to HS or CSB have been resolved by Reza Khayat’s group and, interestingly, are discontinuously distributed on the capsid surface [25]. Based on our analysis of electrostatic properties, PCV4 has 14 polar residues on the capsid surface, including seven positively charged residues (R and K) and eight polar amino acid residues (N and Q) (Figure 4A), with more polar residues than PCV2 (Figure 4B). In general, residues on the outer surface of the Cap are locations where the Cap interacts with the environment (e.g., cell receptors). Therefore, a better environment is created for receptor binding by hydrogen bonds, increasing its polarity by increasing polar residues on PCV4 capsid surface. Although the receptor and cell entry mechanism of PCV4 are unknown, we reported that PCV4 virus-like particles (VLP) can efficiently enter porcine cell lines (PK15 and 3D4/21) [23]. Thus, we hypothesized that PCV4 may have evolved with increasing polar residues via nonspecific interactions of the virus with negatively charged HS, CSB or perhaps other cell membrane receptors during PCV4 infection.

## Epidemiology of PCV4 and clinical diseases associated with this virus

Since the first discovery of PCV4 in the Hunan province of China, there have been several serological surveys and molecular epidemiology reports. PCV4 was detected in pigs of various age groups and in several tissues, as co-infections with other pathogens, and with a variety of clinical signs, as summarized in Table 1. To date, PCV4 genomic DNA has been detected in pigs in several provinces in China, as well as in South Korea, with positive rates ranging from 1.6 to 45.39% [4, 10]. PCV4 can infect almost all ages of pigs, including sucking, weanling, and fattening pigs, as well as sows and fetuses [4, 10, 11, 15]. Additionally, PCV4 genome has been detected in sera and other tissues, including heart, liver, spleen, lung, kidney, lymph node, tonsil, intestine, and brain. Thus, we inferred that PCV4 has a wide tissue tropism, facilitating both horizontal and vertical transmission. Interestingly, PCV4 remains geographically confined to Asia (China and Korea), but it has not been detected in Europe. Several animals (mice, dogs and cattle) may serve as reservoirs for PCV, with potential for cross-species transmission of PCV to swine [26]. However, currently, it is not clear whether intermediate hosts are involved in transmission of PCV4 to swine herds. Therefore, further monitoring and a better understanding of the molecular epidemiology of PCV4 in potential intermediate hosts should provide new insights into the limited PCV4 geographic distribution and assist in controlling PCV4 transmission.

**Table 1:** **Detection of PCV4 in clinical samples**.

Region	Host	Sample	Year	Clinal signs	Positive rate	Co-infections	Reference
Hunan	Pigs	Serum, lung, spleen and kidney	2019	Respiratory disease, diarrhea, PDNS	12.8% (24/187)	/	[3]
Henan	Sow, sucking, weaning, grower, aborted fetuses	Heart, liver, spleen, lung, kidney, brain, intestine, lymph node, serum	2012–2020	Neurological symptom, PDNS, diarrhea, enteritis, PMWS, respiratory symptom, abortion, encephalitis	45.39% (69/152)	Both PCV2, 3, PRV, PEDV, and PRRSV	[4]
Inner Mongolia	Healthy sows, nursery and fattening pigs	Serum	2016–2018	/	1.6% (27/1683)	PCV2 or PCV3	[10]
Henan and Shanxi	Suckling, weanling pigs, fetuses	Brain, heart, liver, spleen, kidney, lung, lymph nodes, small intestines	2018–2019	Respiratory symptom, diarrhea, neurological symptom, PMWS	25.4% (16/63)	Both PCV2, 3 and PRV; Both PCV2 and PRV	[11]
Guangxi	Pigs	Serum	2015–2019	Respiratory disease, PDNS	5.1% (13/257)	PCV2 or PCV3; Both PCV2 and PCV3	[12]
Anhui	Pig	Lung, spleen, kidney, duodenum	2019	Skin diseases	10.71% (18/168)	PCV2 or PCV3; Both PCV2 and PCV3	[13]
Jiangsu	Pig	Lymph node, tonsil, lung, kidney and liver	2018–2019	Dead pigs	3.33% (4/120)	Both PCV1, 2, 3	[14]
South Korea	Sow, aborted fetuses, suckling, weaned, growers	Lung, spleen, heart, kidneys	2019–2020	Healthy suckling pigs, dead pigs, aborted fetuses	3.28% (11/335)	/	[15]
Korea	Pigs	Tissues, serum, oral fluids	2020–2021	Diseases pigs	39.3% (57/145)	/	[16]
Henan	Pigs	Liver, spleen, kidney, lung, lymph nodes, intestine, serum	2020–2021	/	33.33% (45/133)	PCV2	[18]

/ no information

In a serological survey of 1790 serum samples collected from pigs in 17 provinces of China between 2008 and 2020, overall seroprevalence of PCV4 was 43.97% [5]. PCV4 Rep-specific antibodies were detected in sera from pigs of various ages, with the highest prevalence (67.8%) in sows, and the earliest evidence from a sample collected in 2008 [5]. In another serological survey of 1048 pig serum samples collected from Jiangsu province of China between 2018 and 2021, 3.44% of samples had PCV4 Cap-specific antibodies [27]. Although these serological studies provided insights into the prevalence of PCV4 in pigs, dynamics of viremia and antibody responses to PCV4 infections in swine herds require further investigation.

Pathogenesis of PCV4 is not yet well established. So far, PCV4-associated clinical manifestations have been described in various age groups of pigs, including PDNS and respiratory symptoms [3, 4, 12], postweaning multisystemic wasting syndrome (PMWS), neurological symptoms and diarrhea [4, 11], enteritis and encephalitis [4], and skin disease [13]. In addition, PCV4 may also cause reproductive dysfunction via vertical transmission, boosting its prevalence in aborted fetuses and sows, and implying an association with reproductive failure [4, 15]. Of note, co-infections of PCV4 with other PCV are common in swine herds. PCV2 causes PMWS and PDNS [1], and PCV3 may be associated with PDNS and reproductive failure [2, 28]. Co-infections of PCV4 with PCV2, PCV3, or both, have been described in pigs with clinical signs of PMWS, PDNS and reproductive failure [4, 11, 12]. Moreover, the correlation between PCV4 virus titers and clinical manifestations in pigs may be helpful to elucidate PCV4 pathogenesis. Two groups demonstrated that PCV4 produces a moderate viral load [15, 18]. Furthermore, the positive rate and viral DNA loads of PCV2 and PCV4 are higher in spleen and lymph nodes than other tissues [18], indicating PCV2 targets lymphoid tissues and causes lymphocyte depletion, consistent with previous studies [1]. Based on the similar immune tissue tropism between PCV4 and PCV2, PCV4 may act as a co-factor, or independently cause subclinical infections in pigs with moderate viral loads. In addition, there are reports of co-infection of PCV4 with pseudorabies virus (PRV), porcine epidemic diarrhea virus (PEDV) and porcine reproductive and respiratory syndrome virus (PRRSV) in pigs with neurological symptoms, encephalitis, diarrhea, and enteritis [4, 11]. Based on current evidence, PCV4 is often detected in co-infections with other viral pathogens that make it difficult to elucidate the role of PCV4 in disease pathogenesis. Thus, isolating this virus or rescuing PCV4 using infectious clones will provide new insight. Very recently, PCV4 was successfully rescued from an infectious clone of infected piglets, leading to visible pathological changes in several organs in PCV4-inoculated piglets [29]. In addition, PCV4 viremia, PCV4-specific antibody and up-regulated cytokines were detected in PCV4-inoculated piglets. These results implied that PCV4 is pathogenic in piglets, which may pose a great threat to the swine industry.

PCV4 can be detected with several diagnostic methods, including polymerase chain reaction (PCR), real-time PCR, and indirect enzyme-linked immunosorbent assay (ELISA). Nevertheless, immunochemistry or in situ hybridization should be used to detect virus-specific antigen or viral RNA expression in various tissues and its associated histopathology. Applying these diagnostic techniques to PCV4 would promote the understanding of the pathogenesis and molecular epidemiology of this emerging porcine circovirus.

## Future perspectives

As four distinct PCV are circulating in swine herds worldwide, exploring serologic cross-reactivity of PCV4 with other PCV is important to establish reliable serological diagnostic methods. Two studies demonstrated that the established PCV4 ELISA, based on Rep or Cap, had no serological cross-reactions with positive sera of other PCV [5, 27]. Moreover, our previous study also demonstrated an absence of cross-reactions between PCV4 and either PCV2 or PCV3 [23]. VLP, morphologically and immunogenically similar to their native viruses, are widely used for novel vaccine design and serological diagnosis. VLP, assembled by a number of subunits (e.g., PCV2 VLP are assembled from 60 Cap subunits), contain multivalent epitopes (i.e., conformational epitopes), with higher avidity to antibodies compared to the subunit protein. Therefore, VLP are advantageous as an antigen for serological diagnostic tests. PCV4 VLP, prepared in *E. coli*, were highly immunogenic in vivo, and can be used as candidate vaccine and research tools for PCV4, as we reported [23]. Thus, PCV4 VLP have much potential for the development of serological diagnostics for PCV4. In addition, Misinzo et al. used PCV2 VLP to investigate the effects of HS or CSB on binding of PCV2 VLP to host cells [24]. Due to the lack of an efficient cell cultivation method for PCV4, the use of PCV4 VLP has great potential to further characterize this virus. That PCV4 VLP can be produced with high purity and yields make them a preferred tool instead of virus to study cellular receptors during early stages of virus attachment.

As co-infections of PCV4 with other PCV are common in swine herds, there is rationale for developing multivalent PCV combinations to protect against these co-infections of PCV. Based on sequence alignment, PCV4 had low identities (<50%) with three other PCV Reps and Caps. Nevertheless, PCV4 had identities of ~43–48% with PCV1 or PCV2 Reps and Caps, and it is important to analyze the conserved epitopes among the 4 distinct PCV for vaccine design or antiviral strategies against PCV co-infections; However, whether these PCV share conserved epitopes requires further investigation.

## Conclusion

PCV4 is a potential pathogen associated with several clinical signs or syndromes, including PDNS, respiratory or enteric diseases and reproductive failures. This virus has been detected in almost all porcine tissues, particularly in spleen and lymph nodes. Moreover, co-infection of PCV4 with other PCV or pathogens is common in pigs. Therefore, we should focus on monitoring the prevalence and co-infection of PCV4 with other pathogens, as well as continue to closely monitor dynamic changes in genetic diversity and molecular epidemiology of dominant PCV4 strains. Meanwhile, as the exact pathogenesis of PCV4 remains to be elucidated, virus isolation of PCV4 in clinical samples or PCV4 rescued using infectious clones should provide more insight to better elucidate PCV4 pathogenesis and associated diseases.

## Data Availability

The data that support the findings of this study are available on request from the corresponding author.
